# The Treatment of Wounds Controversy

**Published:** 1916-01-08

**Authors:** 


### The Hospital

The Workers' Newspaper of Administrative Medicine and Institutional Life, Administration, National Insurance and Health.

No. 1543, Vol. LIX. SATURDAY, JANUARY 8, 1916. PRICE ONE PENNY.

### THE TREATMENT OF WOUNDS CONTROVERSY.

The controversy which has arisen in reference to the treatment of wounds as these offer themselves in the present war may be attributed in part to certain new experiences and in part to differences of temperament which are inherent in human nature. The novelties of experience relate rather to degree than to kind. Infected and septic wounds are certainly no new thing, but never have they appeared in such numbers and never have the complications which sepsis involves been so universal. The explanation is that, at least as far as the Western front is concerned, the fighting is taking place in regions where there has lived a large population of human beings and other animals, with the necessary consequence that the soil is crowded with micro-organisms, and that certain of these find in wounds a favourable habitat. The conditions of trench warfare, with the inevitable soiling of the men's clothing, and the almost stationary position of the contending armies, offer contributions to the same end. The latter circumstance means multiplied opportunities for further contamination of the soil, and infected clothing affords a ready chance for an infected wound. This in a few words is the series of events which have determined the problem that the military surgeon has to face. The septic wound is not the exception, but the rule, and the pressing and immediate question is as to the best way of dealing with it. In essence, as we have already said, the position is not a new one, but it has become prominent because the surgeon in civil practice has for years used skilful endeavours to prevent infection of the wounds which he inflicts and has in a very large measure succeeded. But now what Art managed to avoid save in a minority of instances, Nature—for the soil of Northern France comes within the title—makes almost universal, and it is no great matter for wonder that different authorities advise different policies in an attempt to deal with the actual situation.

Another factor in determining the treatment of wounds controversy is one which makes its appearance in many scientific debates and practical discussions. Men differ from one another in the temper of their minds and in their mental outlook, and inevitably these differences react on the attitude which they assume to every subject which engages their attention. In some there is a strong inclination to abide in the old ways and to look doubtfully on any proposal which would disturb use and wont, whilst on others novelty exercises an almost invariable charm, and even a chequered experience hardly dims the glow of confident anticipation. There is no difficulty in finding illustrations of both these dispositions in the present instance, and each must be admitted as not without value. The ideal is to combine them in due proportion, and this end, not easy to obtain in any circumstances, is doubly difficult when a controversial atmosphere has once been established. In such circumstances, whoever else suffers from the dispute, the person who recalls the wisdom of the middle way is not likely to have an easy time. He is regarded with suspicion by both camps, and receives good words from neither. Yet, if history is to be trusted, he is often justified by the event, and when the fog of controversy has cleared away his position is found to be the wise one, and even the necessary one. It is, therefore, no rash thing to say that this experience may be repeated in connection with the present issue. This is not to suggest that the enthusiasm of the innovators is founded on a delusion and ought to be repressed. Without such an influence new ideas might fail to secure attention, and we must be tolerant, even to exaggerations, for the sake of the germ of truth which they often contain. Equally, on the other hand, is patience necessary towards those who insist with unfailing reiteration on the traditions of the elders, for without these the chaos of revolution would be a daily event. On these principles it is necessary to give heed both to those who tell us that antiseptics as applications to wounds, at least to wounds inflicted in war, are useless, and also to others who push aside laboratory observations as unnecessary for the art of practical surgery. There are incidents which go to show that neither of these extreme positions can be defended with success. Yet without the statement of each there is a risk that the equally misleading alternative may gain undisputed sway.

Fortunately the debate is not to be left in the hands of the enthusiasts either in one direction or the other. There are, in spite of the storm, some voices which remain steady and which are directed by an unemotional judgment. In this respect particularly much attention will be paid to the recent summary of personal experience given by Sir Anthony Bowlby in his Bradshaw lecture. To the more fervent apostles of the hour it may seem to savour of a prehistoric age to talk about "velvety granulations" and "laudable pus," and of "silver nitrate" and "zinc sulphate" as beneficial applications in the treatment of wounds. Yet these recognitions and commendations are urged not by one who has no experience of the new proposals and who merely stands fast in an attachment to a closed decision. On the contrary, Sir Anthony has tested the hypertonic salt solution and has recognised its values. Of these he speaks frankly and with no stinted praise. But not less does he recognise that a single method and a single agent cannot and do not meet conditions which vary within wide limits, and which call in their various manifestations for adjustment directed by clinical experience. The excellences of the new suggestions are allowed and welcomed, but they do not demand or even justify a compendious discarding of agents and agencies which have a well-established tradition to their credit. After listening to the advocates as is meet and right, the time arrives for the summing up of the judge. Even this may be modified by time, but for present purposes its value will be recognised as of high practical importance.

